# Clinical outcomes of the single-stage revision technique in conversion total hip arthroplasty after failed femoral neck fractures: a two-year follow-up study

**DOI:** 10.1186/s42836-025-00364-5

**Published:** 2026-01-19

**Authors:** Jiankang Pan, Yongqiang Sun, Shuailei Li

**Affiliations:** Department of Arthroplasty Revision, Henan Luoyang Orthopedic Hospital (Henan Provincial Orthopedic Hospital), Zhengzhou, Henan, 450000 China

**Keywords:** Unexpected positive intraoperative culture, Periprosthetic joint infection, Single-stage revision

## Abstract

**Background:**

Conversion to total hip arthroplasty (THA) is associated with higher rates of infection. The purpose of this study is to determine whether applying the surgical technique of single-stage revision can effectively reduce the infection rate of conversion THA after failed femoral neck fractures.

**Methods:**

A retrospective cohort study was conducted on patients who underwent conversion THA after failed femoral neck fracture between January 2019 and December 2022, with a minimum follow-up of 2 years. From January 2019 to March 2020, patients undergoing conversion THA were managed as a primary procedure without synovial fluid culture (Group A). From April 2020 to December 2022, patients undergoing conversion THA were managed with the single-stage revision technique and routine intraoperative synovial fluid culture (Group B). The patients in Group B were matched 1:1 to patients in Group A. Unexpected positive intraoperative culture (UPIC) results were recorded, and PJIs were monitored during the minimum 2-year follow-up period.

**Results:**

As intraoperative cultures were only performed in Group B, the unexpected positive intraoperative culture (UPIC) results presented were solely for Group B. Among the patients in Group B who underwent conversion THA, 91% had no UPIC (90 of 99), 7% had a single (either anaerobic bottle or aerobic bottle) UPIC (7 of 99), and 2% had two (both anaerobic and aerobic bottles) UPICs (2 of 99). In Group A, 7 patients (7/99, 7%) experienced PJIs, compared to 1 patient (1/99, 1%) in Group B, showing a significant difference between the two groups (*P* = 0.030).

**Conclusion:**

As a novel method for conversion to THA after failed femoral neck fracture, the single-stage revision technique is potentially associated with a lower incidence of PJI. Further evaluation of this technique in larger comparative series is warranted.

Video Abstract

**Supplementary Information:**

The online version contains supplementary material available at 10.1186/s42836-025-00364-5.

## Introduction

The number of femoral neck fractures continues to increase as the population ages. Treatments for such fractures depend on the patient’s age, medical comorbidities, and type of fracture. If the fracture unites, internal fixation is usually the first-line treatment for these fractures, and outcomes can be favorable. However, nonunion and avascular necrosis are concerns, potentially leading to conversion to total hip arthroplasty (THA) as a salvage treatment.

Several studies demonstrate that conversion to THA for failed internal fixation has higher infection rates when compared to primary THA [1, 2], particularly in cases with unexpected positive intraoperative culture (UPIC). The rate of UPIC during conversion THA ranges from 12 to 18% [3, 4], and the postoperative periprosthetic joint infections (PJI) as high as 7%–18% at 1-year follow-up [2, 3].One study demonstrated that conversion THAs are more similar in clinical and 30-day postoperative characteristics to revision THAs than to primary THAs [5]. Currently, there are no guidelines or recommendations regarding hip synovial fluid cultures in conversion THA. It is challenging to acquire an adequate hip synovial fluid sample for culture preoperatively, and intraoperative culture results require a waiting period of several days, thereby delaying the initiation of targeted antibiotic therapy.

Single-stage revision has emerged as a promising option for the treatment of PJI, with infection control rates as high as 94.3% [6–8]. The success of this technique relies on extensive and meticulous debridement of all hardware and infected tissues [6, 9]. Given the promising outcomes of single-stage revision for PJI, this study aimed to determine whether the application of this technique would effectively reduce the rate of PJI in conversion THA.

## Patients and methods

We conducted a retrospective review of patients at our institution who underwent conversion THA after failed femoral neck fracture as a salvage procedure following prior internal fixation for femoral neck fractures from January 2019 to December 2022.

Patients who underwent conversion THA with prior internal fixation for a femoral neck fracture were eligible for inclusion. Serum erythrocyte sedimentation rate (ESR) and C-reactive protein (CRP) are first-line serum markers used to rule out infection. Consequently, patients with ESR > 20 mm/h or CRP > 10 mg/L were excluded. Additional exclusion criteria were patients with inflammatory conditions (e.g., rheumatoid arthritis or systemic lupus erythematosus). Preoperative hip joint aspiration for culture demonstrates high sensitivity and specificity for excluding infection in patients with pre-existing hardware [10]. So, positive pre-operative cultures or synovial fluid analysis indicated occult infection were also excluded.

Preoperative hip joint aspiration was performed for all patients as follows [11]. When more than 0.5 mL of hip synovial fluid was obtained, the aspirated hip synovial fluid was directly injected into BACT/ALERT anaerobic and aerobic culture bottles for 9 to 11 days [6]. When less than 0.5 mL of synovial fluid was aspirated, priority was given to synovial fluid analysis. For culture, repeated joint aspiration was administered to these patients. If the aspiration site was still a “dry tap” case, 10 mL of saline solution was injected and re-aspirated. This solution was then divided equally and inoculated into the abovementioned bottles for 9 to 11 days.

**Fig. 1:**
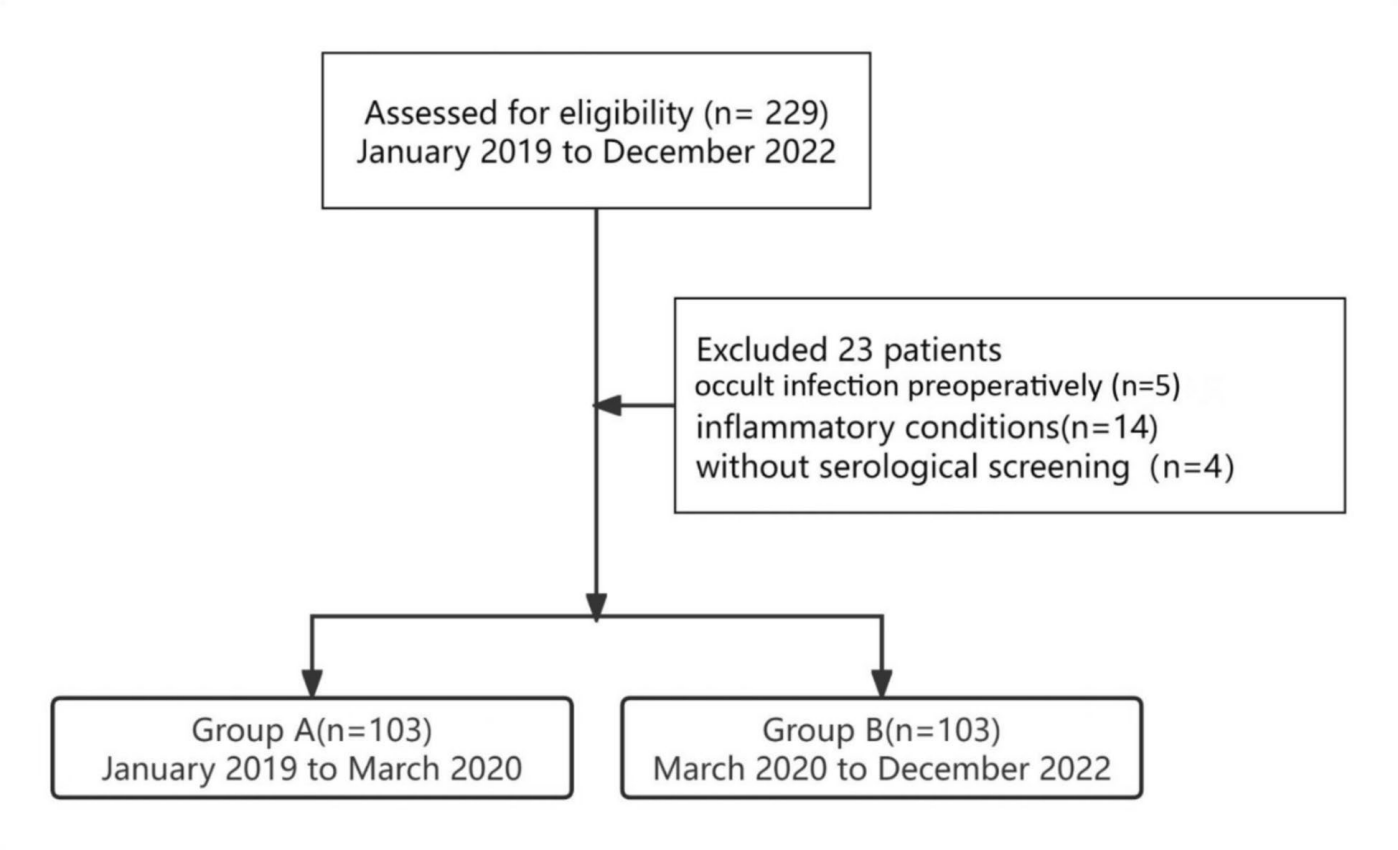
This flowchart represents eligible patients who underwent conversion THA following prior internal fixation for femoral neck fractures

Conversion THA carried a high risk of PJI, yet no specific guidelines existed for such a special group. From January 2019 to March 2020, patients who underwent conversion THA after failed femoral neck fracture were treated as those undergoing primary procedure and did not receive intraoperative synovial fluid culture (Group A). From April 2020 to December 2022, they were managed with a single-stage revision technique, with routine intraoperative synovial fluid culture performed for all patients (Group B). Of the 229 patients assessed for eligibility, 10 patients in Group A, and 13 patients in the Group B met the exclusion criteria and were excluded from the study (Fig. 1), The remaining 206 patients were eligible for subsequent 1:1 propensity score matching using the nearest-neighbor method based on the following covariates: age, gender, body mass index (BMI), smoking status, comorbidities, interval to conversion, type of internal fixation, indication for conversion THA, and rate of dry tap.

All the eligible conversion THAs after failed femoral neck fracture were performed with a posterior approach by the same senior surgeon (Shuailei Li), who is experienced in hip revision and infection treatment. The surgical technique used for Group A was as primary procedure. The surgical technique used for Group B was a single-stage revision for hip PJI. The failed internal fixations were removed, and intraoperative synovial fluid aspiration was performed under direct vision. Synovial fluid samples were sent to the microbiology laboratory for cultures as soon as possible. Additionally, five intraoperative periprosthetic tissue samples obtained around the internal fixations were sent for histologic evaluation. Prophylactic antibiotics were administered intravenously (IV) until intraoperative cultures were sent.

Debridement was performed, involving the removal of all fibrous tissue and synovial tissue. After that, the acetabulum and femur were prepared. To prevent varus of the femoral stem, the sclerotic bone on the lateral side of the greater trochanter should be removed with an osteotome, with intraoperative fluoroscopy utilized to ensure accuracy. The operative field was then thoroughly irrigated with 3 L of saline, followed by complete irrigation with 100 mL of 3% hydrogen peroxide. Subsequently, the area was soaked in 400 mL of 0.1% betadine for 15 min, after which pulsed irrigation with 3 L of sterile saline was performed. The operative field was then re-sterilised and rewrapped. During this process, the surgical team donned and gloved, and the entire set of instruments was exchanged.

0.5 g of vancomycin was poured into the medullary cavity and the base of the acetabulum, followed by the placement of calcium sulfate beads loaded with vancomycin powder (10:1 ratio) deep into the fascia [12]. All patients underwent cementless total hip arthroplasty, and no drainage was used for any patient.

Standard perioperative antimicrobial prophylaxis (2 g of intravenous cefazolin) was administered in all cases. Extended antibiotic prophylaxis was administered with a low threshold if either of the following criteria was met: the culture was not deemed contaminated upon microbiological assessment using the previously described algorithm (based on time to positivity; the number of positive blood cultures; pathogenicity and virulence; each patient’s clinical history; leukocyte count; body temperature; results from other sites; radiographic data; histopathologic findings and current status) [13], or there was histologic evidence of infection (defined as greater than five neutrophils per high-power field in periprosthetic tissue samples) [14]. These patients were then treated with a 6-week intravenous course of pathogen-sensitive or empirical antibiotics.

Patient data, including synovial fluid cultures, hemoglobin reduction [15], and surgical time were also documented. The Harris Hip Score (HHS) was used to evaluate patient-reported outcomes, and data were collected preoperatively and postoperatively. Clinical signs or symptoms of infection, such as pain at rest and draining sinus tracts, were closely monitored. Removal of the components was also monitored during the follow-up period. All the patients were monitored for a minimum of 2 years of follow-up. PJIs were diagnosed according to the Musculoskeletal Infection Society (MSIS) criteria [16].

### Statistical analysis

Univariate analysis was performed using the Chi-square test. Continuous variables were compared using the *t*-test. A Kaplan Meier survival curve was used to evaluate failure due to infection and removal of prosthetic components due to any indication. Cox regression hazard ratio (HR) analysis was used to identify risk factors for PJI. Spearman’s rank correlation was calculated to evaluate the correlation between UPIC and histologic results. Statistical tests were conducted using Graphpad Prism 10.1, with significance defined as *P* < 0.05.

## Results

All patients were followed up for a mean of 3 years (range, 2–4 years), and no one was lost before the minimum of 2 years. The two groups were matched in demographic details, and there were no significant differences in patient demographics between the two groups shown in Table 1.

**Table 1:** Characteristics of the study population before and after matching

	**Before propensity score matching**			**After propensity score matching**		
	**Group A**	**Group B**	***P*-value**	**Group A**	**Group B**	***P*-value**
Number of patients	103	103		99	99	
Mean age, y (SD)	66.8(4.2)	66.7(5.0)	0.29	67.1(4.1)	66.4(5.0)	0.865
Gender (Male), *n* (%)	53(51.4)	48(46.6)	0.49	51(51.4)	48(46.6)	0.67
BMI, kg/m^2^ (SD)	25.8(1.2)	25.6(1.0)	0.23	25.6(1.2)	25.7(1.1)	0.69
Smoking status, *n* (%)						
Smoker	30(29.1)	27(26.2)	0.64	28(29.1)	26(26.2)	0.75
Comorbidities, *n* (%)						
Type II diabetes	12(11.6)	13(12.6)	0.83	10(10.1)	10(10.1)	0.867
Peripheral vascular disease	2(2)	0		-	-	
Gout	10(9.7)	8(7.7)		6(6.1)	8(8.1)	
COPD	8(7.7)	9(8.7)		5(6.1)	7(7.1)	
Interval to conversion in months (SD)	48.9(5.8)	50.3(5.5)	0.07	49.8(5.7)	50.0(5.2)	0.428
Type of internal fixations, *n* (%)						
Angle-fixed devices	14(14.6)	12(11.6)	0.6	12(12.1)	12(12.1)	0.913
CCS	76(77.7)	73(70.1)		75(75.6)	73(73.7)	
CCS with plates	13(13.6)	18(17.5)		12(12.1)	14(14.1)	
Indication for conversion THAs, *n* (%)						
Avascular necrosis	42(40.1)	36(34.9)	0.67	40 (40.1)	36(34.9)	0.839
Non-union	51(49.5)	55(53.4)		50(49.5)	53(53.4)	
Implant failure or cut out	10(9.7)	12(11.6)		9(9.7)	10(11.6)	
Dry tap, *n* (%)	68(66.0%)	72(69.9%)	0.13	68(68.7%)	70(70.7%)	0.532

*SD* standard deviation, *BMI* body mass index, *COPD* chronic obstructive pulmonary disease, *CCS* cannulated compression screws, *THA* total hip arthroplasty

Significant differences were observed between Group A and Group B in terms of average surgical time and hemoglobin reduction (54.7 ± 7.7 min vs. 81.6 ± 6.3 min; *P* < 0.01) and (13.1 ± 1.9 g/L vs. 26.2 ± 2.9 g/L; *P* < 00.01), respectively. No patient required a blood transfusion during treatment. The mean pre-operative HHS improved significantly at the last follow-up in both groups (Table 2).

**Table 2:** Patient hip functional outcomes

	**Preop HHS (SD)**	**Last follow-up HHS (SD)**	***P*-value**
Group A	56.0 (13.2)	95.2(14.5)	< 0.001
Group B	54.7 (15.1)	94.1(18.7)	< 0.001
*P*-value	0.520	0.644	

*Preop* preoperative, *HHS* Harris Hip Score, *SD* Standard Deviation

Of the patients who underwent conversion THA in Group B, 91% (90 of 99) had no UPIC, 11% (11 of 99) had a single (anaerobic bottle or aerobic bottle) UPIC, and 2% (2 of 99) had two (both anaerobic and aerobic bottles) UPICs. Among the cases with a single UPIC, 4 patients were considered contaminated and received only antibiotic prophylaxis. The remaining 7 patients were treated with a 6-week intravenous course of pathogen-sensitive antibiotics. 2 patients had two UPICs, and both were treated with a 6-week intravenous course of pathogen-sensitive antibiotics (Table 3). A total of 9 samples from 8 patients had positive histologic evidence of infection (Table 3), and all the patients received a 6-week intravenous course of pathogen-sensitive or empirical antibiotics. The duration between the administration of prophylactic antibiotics and pathogen-sensitive or empirical antibiotics was 7 days (range, 4–9 days). A positive correlation was found between UPIC and histologic result (*r* = 0.834; 95%CI, 0.720–0.864; *P* < 0.001).

**Table 3:** Summary of unexpected positive intraoperative cultures or positive histologic evaluation in group B

Case	Internal fixation	Dry tap	Type of UPIC	Organism	Number of positive histologic evaluations
1	CCS	Yes	2	Staphylococcus epidermidis	1
2	DHS	No	1	Escherichia coli	0
3	DHS	Yes	1	Staphylococcus aureus	0
4	CCS	Yes	1	Candida albicans	0
5	CCS	No	1	Enterococcus faecium	1
6	CCS with plates	Yes	2	CoNS	0
7	CCS	No	1	Citrobacter koseri	2
8	CCS	Yes	1	Staphylococcus hominis	0
9	CCS	Yes	1	MRSE	1
10	CCS	Yes	0	Negative	1
11	DHS	Yes	0	Negative	1
12	CCS	No	0	Negative	1
13	CCS	Yes	0	Negative	1

*UPIC* unexpected positive intraoperative cultures, *CCS* cannulated compression screws, *DHS* Dynamic hip screw, *CoNS* coagulase-negative **s**taphylococcus aureus, *MRSE* methicillin-resistant staphylococcus epidermidis

**Fig. 2:**
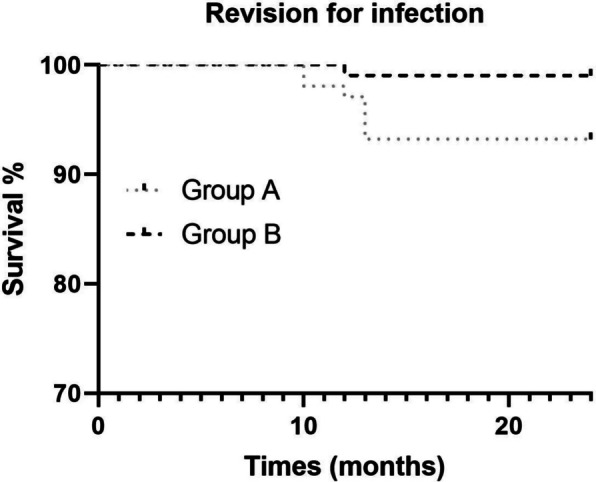
Kaplan–Meier survival curves represent revision for infection

Among the 198 patients, 8 patients experienced PJIs (Table 4), with 7 in Group A (7/99, 7%) and 1 in Group B (1/99, 1%). This difference between the two groups was statistically significant (*P* = 0.030). There were no associations between PJIs and age, gender, BMI, smoking status, co-morbidities, interval to conversion, type of internal fixations, indication for conversion THAs, or rate of dry tap (Table 5). The hazard ratio (HR) for revision due to infection in Group B, compared with that of Group A, was 0.216 (95% CI: 0.05 to 0.86; *P* = 0.031) (Fig. 2). Of the 7 patients in Group A who experienced an infection-related failure 13 months (range, 2–18 months) after surgery, the revisions were 2 patients underwent debridement, antibiotics, irrigation, implant retention (DAIR) to eradicate the infection; 3 patients underwent single-stage revision; and 2 patients underwent two-stage revision. The same patient in Group B (case 6 in Table 3 and case 8 in Table 4) experienced an infection-related failure 14 months following conversion to THA. Intraoperative cultures obtained during the conversion to THA were Coagulase-negative Staphylococcus aureus, while cultures taken during the subsequent revision arthroplasty were Propionibacterium acnes. The infection was managed with a DAIR to eradicate the infection.

**Table 4:** Summary of periprosthetic joint infection cases in both groups

Case	Group	Internal fixation	Organism intra-operative	Organism in revision	Type of revision
1	A	CCS	-	CoNS	single-stage revision
2	A	DHS	-	MRSE	DAIR
3	A	DHS	-	MRSE	single-stage revision
4	A	CCS	-	MRSA	two-stage revision
5	A	CCS	-	CoNS	DAIR
6	A	CCS	-	Staphylococcus epidermidis	single-stage revision
7	A	CCS	-	MRSA	two-stage revision
8	B	CCS with plates	CoNS	Propionibacterium acnes	DAIR

*CCS* cannulated compression screws, *DHS* Dynamic hip screw, *CoNS* coagulase-negative staphylococcus aureus, *MRSE* methicillin-resistant Staphylococcus epidermidis, *MRSA* methicillin-resistant Staphylococcus aureus, *MSSE* methicillin-sensitive staphylococcus epidermidis, *DAIR* debridement, antibiotics, irrigation, and implant retention

**Table 5:** Cox regression hazard ratio survival analysis for risk factors of periprosthetic joint infection in conversion total hip arthroplasty

Variable	HR (95% CI)	*P*-value
Age	1.010(0.8639 to 1.206)	0.5110
Gender (Male)	0.933(0.3760 to 11.43)	0.4402
BMI	0.627(0.2577 to 1.377)	0.3306
Smoking status	1.656(0.2874 to 8.149)	0.6778
Comorbidities	1.183(0.2484 to 7.006)	0.8390
Interval to conversion in months	0.932(0.7952 to 1.074)	0.3482
Type of internal fixations		
Angle-fixed devices	Reference	
CCS	0.221(0.04460 to 1.243)	0.0644
CCS with plates	0.310(0.01475 to 2.682)	0.3264
Indication for conversion to THAs		
Avascular necrosis	Reference	
Non-union	0.391(0.06731 to 1.951)	0.2558
Implant failure or cut out	0.968(0.04632 to 7.779)	0.9786
Dry tap	3.466(0.8007 to 17.81)	0.1024

*HR* hazard ratio, *CI* confidence interval, *SD* standard deviation, *BMI* body mass index, *CCS* cannulated compression screws, *THA* total hip arthroplasty

**Fig. 3:**
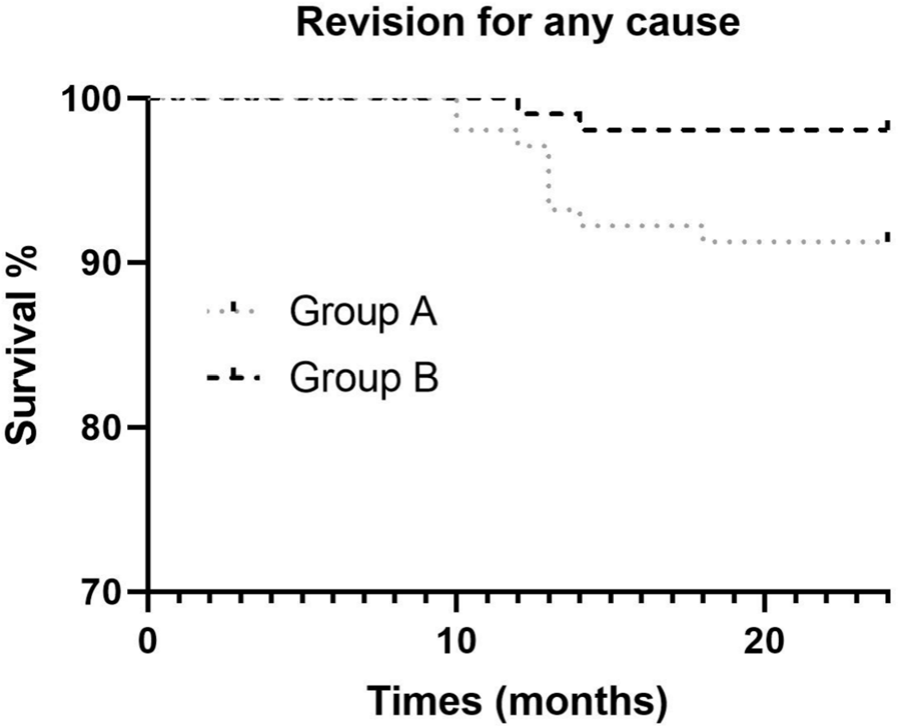
Kaplan–Meier survival curves represent revision for any cause

The HR for removal of prosthetic components due to any indication in Group B, compared with that of Group A, was 0.27 (95% CI: 0.082 to 0.88; *P* = 0.026) (Fig. 3). 2 patients in Group A and 1 patient in Group B underwent components removal due to prosthesis dislocation/instability and leg discrepancy, after 8, 10, and 7 months respectively, with no significant difference observed between the two groups.

## Discussion

Our study aimed to determine whether the single-stage revision technique could reduce the infection rate in conversion THA. Among the 198 patients included in the study, the infection rate in Group A was 7%, while the rate in Group B was 1%, which was significantly higher than that of Group B.

UPIC is not uncommon in conversion to THA. Even though the present study ruled out any suspicion of occult infection in conversion THA preoperatively, 11% of patients still had a single PIC, and 2% had two UPICs, which was consistent with the findings reported by Cichos et al. [3]. In the study, 18% patients had positive intraoperative cultures and 15.7% developed PJI in conversion THA. The positive pre-operative and intra-operative cultures may partially explain the higher infection rate following conversion THA compared with primary THA. Other previous studies also demonstrated that the rate of occult infection ranged from 11.6% to 52% [4, 17] and the rate of PJI ranged from 4.7% to 16% in conversion THA [4, 18, 19].

Several studies stated that conversion to THA was associated with a higher PJI rate than primary THA. Heckmann et al. [5] found that conversion THA had a higher rate of deep incisional surgical site infections (SSI) (0.8% vs 0.3%) and sepsis (1.1% vs 0.3%) compared to primary THA. However, there were no significant differences in deep incisional SSI and sepsis rates between the conversion THA group and the revision THA group. Scott et al. [20] reported that conversion THA was associated with higher rates of PJI compared to primary THA (conversion: 7.7% vs primary:1.4%). Klatte et al. [10] reported a retrospective study assessing whether conversion to THA is associated with an increased risk of PJI at an average follow-up of 3.5 years. In this study, 42% (52/122) of patients underwent preoperative aspiration, with 2 patients (3.8%) exhibiting positive bacterial growth. Additionally, 88.6% (109/122) underwent intraoperative culture sampling, which showed no bacterial growth. No PJI occurred at the latest follow-up, which was lower than that observed in the present study. This outcome may be partially attributed to the fact that 49% primary surgeries were performed for femoral neck fractures and utilized a single-stage technique during conversion THA.

The surgical time in Group B was longer than that of Group A. The debridement process accounted for 27 min, resulting in a total surgical time of 82 min. Prolonged surgical time has been identified as a contributing factor to the development of PJI. A previous study found that surgical times over 90 min were associated with a twofold greater risk of developing surgical site infections than those within 60 min [21]. Given the devastating complication of PJI, the lower PJI rate in Group B weighed against spending the extra time to thoroughly debridement.

To our knowledge, this is the first study to culture hip synovial fluid before conversion THA and to report the technique of single-stage revision in conversion THA. Currently, there are no established guidelines regarding pre-operative and intra-operative cultures and in conversion THA. Intra-operative cultures exhibit a certain delay; how to manage the occult infection in conversion THA poses a significant challenge for clinicians. To rule out occult infection, the lack of accessible hip synovial fluid is a dilemma faced by the surgeon before the operation. In our study, 67% patients were “dry tap” cases. Li et al. [11] reported that re-aspirated saline achieved sensitivity and specificity rates as high as 79.5% and 95.7%, respectively. Fluid samples and histology played an important role in the definition of Periprosthetic Joint Infection [14]. Robert et al. retrospectively reviewed the charts of 49 patients who underwent conversion THA with prior acetabular fracture fixation. In the study, they defined infection as bacterial growth on solid culture medium or any bacterial growth associated with acute inflammation on permanent histologic analysis of tissue. 5 patients had positive intraoperative cultures, and 3 of them had a positive frozen section. In the present study, 9 patients had positive intraoperative cultures, and 4 of them had a positive histology result.

The surgical technique of one-stage revision THA resulted in a sevenfold (7% vs 1%) reduction in PJI risk following conversion THA at minimal additional cost in the present study. This outcome can be partially attributed to extensive and meticulous debridement of all hardware and infected tissues [22, 23]. Another key principle in the surgical treatment of PJI is the adequate local delivery of antibiotics. Ji et al. reported 51 patients with culture-negative PJI who underwent one-stage revision combined with intra-articular vancomycin infusion, achieving an infection control rate of 90.2% [6]. Due to the low rate of positive intraoperative cultures, it was not feasible to leave a suction drain for intra-articular antibiotic infusion during the incubation period for specimens. To achieve high local antibiotic concentrations, our protocol involved delivering vancomycin using calcium sulfate, as described by Kallala et al., who reported a series of 15 patients undergoing revision arthroplasty with an infection rate of 6.6% [12].

### Limitation

This study has several limitations. First, it is a retrospective single-center study rather than a randomized controlled trial; the surgeon was not blinded to the patient’s group assignment, intraoperative synovial fluid culture was only performed in Group B, and patients with positive culture were treated with pathogen-sensitive antibiotics in Group B, which inherently carries limitations and potential biases. Given the devastating complications associated with infection and no established guidelines regarding intraoperative cultures and conversion to THA, antibiotics were administered with a low threshold in these cases. Second, the inability to know the UPIC rate in Group A which is a major confounder. The technique of re-aspirating saline in the hip to rule out occult infection remains controversial; it likely represents the best alternative we have when faced with a high rate of “dry tap”. Previous studies have demonstrated its validity. Third, although histologic analysis combined with positive culture plays an important role in diagnosing PJI following a failed THA, it is unclear whether the proposed criteria apply to conversion THA, potentially leading to antibiotic overuse. Fourth, the potential for detection bias in PJI diagnosis; Fifth, the present study is a single-center surgical technique in the conversion of THA after failed femoral neck fracture. Multi-center studies are needed to validate these findings before the technique can be widely accepted.

## Conclusion

UPIC is not rare in conversion to THA after a failed femoral neck fracture and should be appropriately managed. Conversion THA after failed femoral neck fracture is associated with a high rate of PJI. Despite the aforementioned limitations of the present study, the use of a single-stage revision technique for conversion THA after failed femoral neck fracture might be associated with a lower rate of PJI.

## Data Availability

None applicable.
